# Superconducting Valve Exploiting Interplay between Spin-Orbit and Exchange Interactions

**DOI:** 10.3390/nano12244426

**Published:** 2022-12-12

**Authors:** Alexey Neilo, Sergey Bakurskiy, Nikolay Klenov, Igor Soloviev, Mikhail Kupriyanov

**Affiliations:** 1 National University of Science and Technology MISIS, 119049 Moscow, Russia; 2 Skobeltsyn Institute of Nuclear Physics, Lomonosov Moscow State University, 119991 Moscow, Russia

**Keywords:** spin-valve, superconductivity, nanostructure, spin-orbit interaction, magnetism

## Abstract

We theoretically investigated the proximity effect in SN${}_{SO}$F and SF’F structures consisting of a superconductor (S), a normal metal (N${}_{SO}$), and ferromagnetic (F’,F) thin films with spin–orbit interaction (SOI) in the N${}_{SO}$ layer. We show that a normal layer with spin–orbit interaction effectively suppresses triplet correlations generated in a ferromagnetic layer. Due to this effect, the critical temperature of the superconducting layer in the SN${}_{SO}$F multilayer turns out to be higher than in a similar multilayer without spin–orbit interaction in the N layer. Moreover, in the presence of a mixed type of spin–orbit interaction involving the Rashba and Dresselhaus components, the SN${}_{SO}$F structure is a spin valve, whose critical temperature is determined by the direction of the magnetization vector in the F layer. We calculated the control characteristics of the SN${}_{SO}$F spin valve and compared them with those available in traditional SF’F devices with two ferromagnetic layers. We concluded that SN${}_{SO}$F structures with one controlled F layer provide solid advantages over the broadly considered SF’F spin valves, paving the way for high-performance storage components for superconducting electronics.

## 1. Introduction

The research and design of superconducting (S) spin valves is one of the most-demanded research directions in superconducting electronics (SCE). The International Roadmap for Devices and Systems (IRDS^TM^) [1] puts the investigation of magnetic materials and nanostructures in a list of the active research questions for SCE this year. The ability to control the concentration of superconducting correlations in a certain area via the interplay with ferromagnetic spin ordering is important in the elements of cryogenic memory [2,3,4,5,6], neuromorphic computing systems [7,8,9,10,11,12], and the periphery of quantum computers [13,14,15]. The lack of robust and compact data storage prohibits SCE applications in the field of high-performance computing, where the inherent SCE features such as high energy efficiency and fast operation could otherwise provide a breakthrough.

The principle of superconducting spin valves’ operation is based on the possibility of the effective control of their parameters by changing the magnitude or orientation of the magnetic moments of their ferromagnetic (F) parts [16,17,18,19,20,21,22]. In this way, it is possible to control the critical current of Josephson junctions or the inductance of superconducting multilayer structures [12] by controlling the value of their critical transition temperature to the superconducting state, $T_{C}.$

Despite intensive theoretical [23,24,25,26,27,28,29,30,31,32,33,34] and experimental [35,36,37,38,39,40,41,42,43,44,45,46,47,48,49] study of superconducting triplet spin valves, their practical implementation is still limited. This is due to the need to strictly fix the direction of the magnetization vector of one of the ferromagnets $\vec{M_{1}}$ when changing the direction of the vector $\vec{M_{2}}$ of the other.

In Josephson spin valves, it was suggested to solve the problem of shifting from FF-type control blocks to structures containing only one ferromagnetic layer by complicating the internal structure of the weak coupling region [2,3,50,51,52,53,54] or by taking into account spin–orbit interaction at interfaces and weak link materials (see the review [55] and the references therein).

In spin valve critical temperature control devices, in order to realize this shift, it is necessary to ensure the presence of spin–orbit interaction (SOI) in the structure [55,56,57,58,59,60,61,62,63,64]. Furthermore, the implementation of SOI in the structures reveals the novel class of the spin valve devices, which includes the only ferromagnetic layer. In such devices, it is possible to control the effective influence of the exchange energy by changing the direction of the magnetization vector $\vec{M}$.

**Figure 1:**
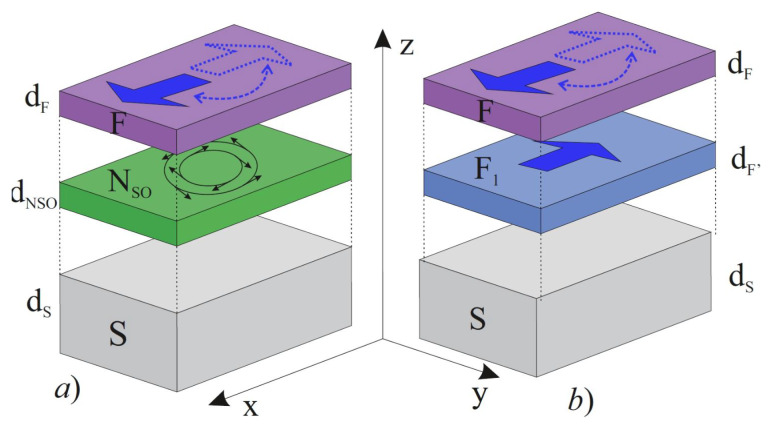
Sketch of (**a**) SN${}_{SO}$F and (**b**) SF’F structures. They consist of a superconducting film (S) with isotropic S-wave pairing potential and ferromagnetic (F) and normal (N${}_{SO}$) layers. It is supposed that there is anisotropic spin–orbit electrons scattering in the N${}_{SO}$ film. The direction of magnetization vector of the upper F layer can be turned in the Oxy plane, while in the middle F${}_{1}$ layer, it is fixed in the direction parallel to the Ox-axis.

In this paper, we considered the trilayer structure superconductor (S)/normal metal with SOI N${}_{SO}$/ferromagnetic metal (F) (SN${}_{SO}$F) (See Figure 1a) and confirmed the spin valve effects, arguing the advantages of SOI devices against the comparison with the broadly considered SF’F structures (See Figure 1b).

## 2. Principle of Operation

Consider a situation where the Rashba [65] or Dresselhaus [66] SOI spin–orbit vector, $\vec{A},$ lies in the N${}_{SO}$ film plane.

(1) $$\vec{A}=A_{x}\vec{n_{x}}+A_{y}\vec{n_{y}}=\left(\beta \sigma_{x}-\alpha \sigma_{y}\right)\vec{n_{x}}+\left(\alpha \sigma_{x}-\beta \sigma_{y}\right)\vec{n_{y}}.$$

**Figure 2:**
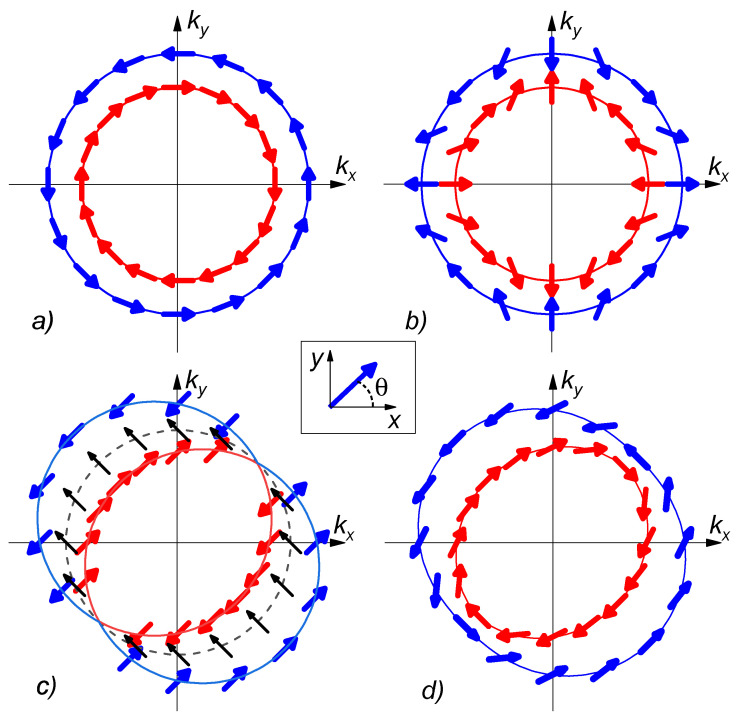
Qualitative picture of the dispersion law in normal metal with different types of spin–orbit interaction: (**a**) Rashba-type α=const, β=0; (**b**) Dresselhaus-type α=0, β=const; (**c**) equally mixed SOI α=β=const; (**d**) mixed SOI with dominance of the Rashba component α=2β=const. The arrows show the spin polarization for a certain sub-band. The inset shows the relation of the magnetization angle θ with the spin majority direction.

Here, α and β are the Rashba [65] and Dresselhaus [66] SOI coefficients, which arise in materials with a violation of spatial (structural) symmetry and with a violation of symmetry in the crystal lattice of the metal; $\sigma_{x}$ and $\sigma_{y}$ are Pauli matrices, which reflect the structure of the components of the vector $\vec{A}$ in the spin space; $n_{x}$ and $n_{y}$ are unit vectors along the Ox- and Oy-axis (see Figure 1). Figure 2 demonstrates a qualitative picture of the dispersion law that takes place in a normal metal with spin–orbit interaction in the presence of the Rashba (Figure 2a) or Dresselhaus (Figure 2b) SOI. The figure shows the spin orientation of the particle with the maximal (blue) and minimal (red) momentum amplitude |k| at a certain energy for a given angle in the plane $\left(k_{x},k_{y}\right)$.

As in the SFF case described above, the spin–orbit coupling again splits the Fermi surface into two sub-bands [67,68] (see Figure 2a,b). The crucial difference is that these sub-bands can no longer be associated with a definite spin. Therefore, as a result of reflections from the SN${}_{SO}$ interface, an incident electron is allowed to be reflected ether as a hole having the opposite direction of the spin and momentum or as a hole with the same spin orientation. It is obvious that this process acts in the direction opposite to that of the spin polarization of electrons in the F layer determined by the magnetization vector, $\vec{M}$, with the direction determined by the angle θ between $\vec{M}$ and the 0x-axis. Both types of SOI are isotropic in the film plane and lead to a decrease in the effective exchange energy induced into the N${}_{SO}$ film [69].

Anisotropy is violated when both types of SOI are present in N${}_{SO}$ (see Figure 2c,d). The violation of the anisotropy appears in momentum *k* as well as in the spin σ space. We concentrate on the latter issue for further discussion, since the orientation of the magnetization of the mutual layer influences the directions of the spin majority.

It can be seen that, at α=β (see the blue line in Figure 2c), the band with the maximal momentum |k| consists of particles with spin directions π/4 and 5π/4 (here and later, we mention the angle between), in contrast to the cases of the true Rashba (a) and Dresselhaus (b) SOI, where the particle spin smoothly depends on the direction of motion. Similarly, the band with the minimal momentum includes particles with the same spin directions. In other words, the dispersion law for such a material consists of two shifted Fermi spheres for particles with spins oriented in directions π/4 and 5π/4.

It matters if the particles with the following spins have different populations. Such a case occurs in the case of the proximity with the ferromagnetic layer with magnetization $\vec{M}$ with non-zero projection on the discussed axis corresponding to rotational angle θ=π/4.

At the same time, the bands corresponding to other spins directions are split weakly. In certain cases of the spin directions 3π/4 and −π/4, the corresponding bands coincide with each other (see the black dashed line in Figure 2c). Such a behavior of the dispersion law provides the most effective spin mixing in the N${}_{SO}$ layer for the spins oriented in directions π/4+πn, while for spins oriented at angle 3π/4+πn, the N${}_{SO}$ layer acts as a conventional normal metal.

In the more general case α≠β (see Figure 2d), the bands are distorted and demonstrate the dependence between the momentum *k* and spin directions. However, even in that case, the splitting in the k-space for particles with spin orientation π/4+πn is still larger than for particles with spin orientation at 3π/4+πn.

This circumstance allows us to foresee that, by changing the ratio between the intensities of the Rashba α and Dresselhaus β SOI or by changing the direction of the magnetization vector $\vec{M}$, it is possible to control the effective exchange energy induced in the film N${}_{SO}$ and, consequently, the $T_{C}$ of SN${}_{SO}$F structures.

## 3. Model

The structures we studied are schematically presented in Figure 1. Below, we will mainly concentrate on the investigation of the processes in the SN${}_{SO}$F structures (see Figure 1a) and then compare the obtained results with the properties of the three-layer SFF (see Figure 1b), which are currently quite well studied both theoretically and experimentally.

The multiple layers presented in Figure 1a consist of a superconducting film (S) with anisotropic S-wave pairing potential and normal (N${}_{SO}$) and ferromagnetic (F) layers. We assumed that dirty limit conditions are fulfilled for all the metals [70,71], F layers have a single domain structure, and their magnetization vector $\vec{M}$ is located in the Oxy plane, resulting in the exchange interaction vector, $\vec{h},$ in the form

$$\vec{h}=h\vec{n_{x}}\cos\theta +h\vec{n_{y}}\sin\theta .$$

Here, *h* is the exchange energy and θ is the angle between the Ox-axis and the magnetization vector direction.

In the N${}_{SO}$ metal, there is a spin–orbit interaction, which we characterize by the vector, $\vec{A},$ also lying in the Oxy plane (1).

With the chosen configuration of vectors $\vec{h}$ and $\vec{A},$ the normal, g, and anomalous, $f_{i},$ i=0,1,2,3, Green’s functions that describe the proximity effect in the three-layer SN${}_{SO}$F structure under study depend only on the coordinate *z* and obey the one-dimensional Usadel equations [71]. Using the differential operator,

(2) $$Df_{i}=\frac{D}{2}g\frac{d^{2}f_{i}}{dz^{2}}-\frac{D}{2}f_{i}\frac{d^{2}g}{dz^{2}}-\omega f_{i},$$

these equations can be represented in the compact form. Here, ω=πT(2n+1) are Matsubara frequencies and *T* is temperature.

In the S layer $\left(0\leq z\leq d_{S}\right)$, both vectors $\vec{A}$ and $\vec{h}$ are zero, and the Usadel equations reduce to:

(3) $$\begin{array}{c} \begin{array}{c} Df_{0}+\Delta g=0,\\ Df_{i}=0,\;i=1,2,3, \end{array} \end{array}$$

(4) $$\Delta \left(\ln\frac{T}{T_{c}}+2\pi T\sum_{\omega >0}^{\infty }\frac{1}{\omega }\right)=-2\pi T\sum_{\omega >0}^{\infty }f_{0},$$

where Δ is a superconductor order parameter.

In the N layer $\left(d_{S}\leq z\leq d_{S}+d_{NSO}\right)$, there is no Zeeman splitting $(\vec{h}=0),$ order parameter Δ=0, and for singlet, $f_{0},$ and triplet, $f_{1,2,3},$ anomalous Green’s functions, we have [56,57,58,72]:

(5) $$\begin{array}{c} \begin{array}{c} Df_{0}=0,\\ Df_{1}-2gD\left(2\alpha \beta f_{2}+\left(\alpha^{2}+\beta^{2}\right)f_{1}\right)=0,\\ Df_{2}-2gD\left(2\alpha \beta f_{1}+\left(\alpha^{2}+\beta^{2}\right)f_{2}\right)=0,\\ Df_{3}-4gD\left(\alpha^{2}+\beta^{2}\right)f_{3}=0. \end{array} \end{array}$$

Finally, in the F layer $d_{S}+d_{NSO}\leq z\leq d_{S}+d_{NSO}+d_{F})$, the Usadel equations have the form:

(6) $$\begin{array}{c} \begin{array}{c} Df_{0}-i\left(f_{1}h\cos\theta +f_{2}h\sin\theta \right)=0,\\ Df_{1}-if_{0}h\cos\theta =0,\\ Df_{2}-if_{0}h\sin\theta =0,\\ Df_{3}=0; \end{array} \end{array}$$

In all the films, the normal and anomalous Green’s functions are coupled by normalization conditions

(7) $$g=\sqrt{1-|f_{0}{|}^{2}+\sum_{i=1}^{3}{\left|f_{i}\right|}^{2}}.$$

To derive it, we used the symmetry relations;

(8) $$\begin{array}{ccc} & f_{0}^{*}\left(-\omega \right) & =f_{0}\left(\omega \right) \end{array}$$

(9) $$\begin{array}{ccc} & f_{i}^{*}\left(-\omega \right) & =-f_{i}\left(\omega \right),\;i=1,2,3, \end{array}$$

which are valid in the absence of a supercurrent in the structure [73].

At the free boundaries of the multilayer, (z=0), $(z=d_{S}+d_{NSO}+d_{F}),$ the anomalous Green’s functions obey the conditions:

(10) $$\frac{d}{dz}f_{i}=0,\;i=0,1,2,3,$$

which guarantee the absence of a current across these interfaces.

At the SN $\left(z=d_{S}\right)$ and NF $\left(z=d_{S}+d_{NSO}\right)$ interfaces, the anomalous functions are coupled by the Kupriyanov–Lukichev boundary conditions [74], which are valid if these boundaries are not magnetically active [75]. Assuming that the decay length ξ and conductivity, ρ, of the materials are the same, we can write the conditions in the form

(11) $$\gamma_{B}\left(g_{l}\frac{d}{dz}f_{l}-f_{l}\frac{d}{dz}g_{l}\right)=g_{r}f_{l}-f_{r}g_{l}.$$

which is valid for each number i=0,1,2,3, and connect the functions $f_{i}$ and $g_{i}$ defined on the left (subindex *l*) and right (subindex *r*) sides of the interfaces. Suppression parameter $\gamma_{B}=R_{B}/\rho \xi ,$ $R_{B},$ is the specific boundary resistance of the interfaces.

To calculate the spatial variations of Δ(z) and $f_{i}\left(z\right),$i=0,1,2,3 inside the multilayers for a set of their geometrical and materials parameters, we developed the numerical code for solving the boundary problem (3)–(11). Below, when analyzing the proximity and spin valve effects in structures, we will track the value of the pair potential $\Delta \left(0\right)=\Delta_{S}$ on the free surface of the superconductor (z=0). This gives us a clear and quantitatively rigorous criterion that allows us to draw conclusions about the degree of influence of magnetic and spin–orbit interactions on the magnitude and presence of superconducting correlations in the structures under study.

## 4. Proximity Effect in SN${}_{\mathrm{SO}}$F Structures

**Figure 3:**
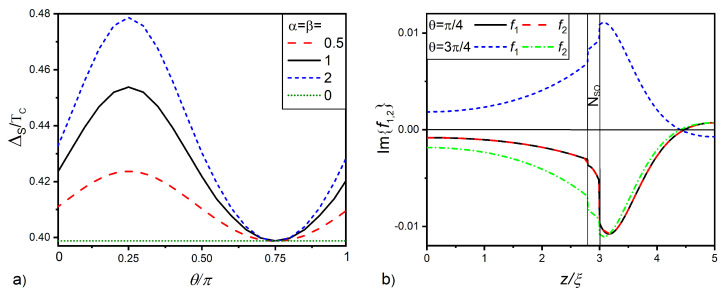
(**a**) Magnitude of the pair potential at free S layer surface $\Delta_{S}$ versus angle θ between the $\vec{h}$ and $\vec{n_{x}}$ directions. (**b**) Spatial distributions of the imaginary parts of anomalous Green’s functions $f_{1}$ and $f_{2}$ calculated for θ=π/4, θ=3π/4, and α=β=1. All calculations were performed for $d_{S}=2.8\xi$, $d_{N_{SO}}=0.2\xi$, $d_{F}=2\xi$, $h=20T_{C}$, $T=0.5T_{C}$, and $\gamma_{B}=0.3$.

**Figure 4:**
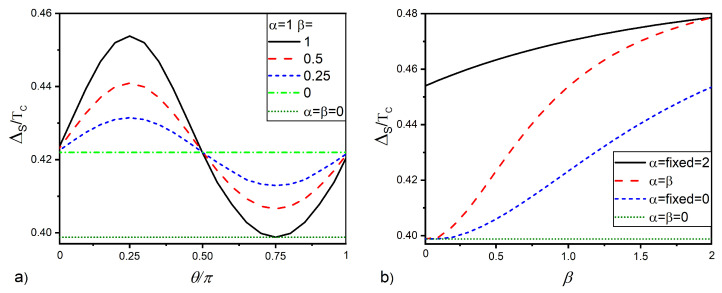
Pair potential $\Delta_{S}$ at the free surface of the S layer versus (**a**) F layer magnetization angle θ for a certain set of SOI coefficients and (**b**) versus the Dresselhaus SOI strength β at θ=π/4 and at different values of the Rashba SOI strength α. Other parameters of the SN${}_{SO}$F structure are: $d_{S}=2.8\xi ,\;/,$ $d_{N_{SO}}=0.2\xi ,$ $/,\;d_{F}=2\xi ,\;h=20T_{C},\;/,\;T=0.5T_{C},\;/,\;\gamma_{B}=0.3$.

The results of the calculations carried out at $T=0.5T_{C}$ and $\gamma_{B}=0.3$ are shown in Figure 3 and Figure 4.

Figure 3a gives the magnitude of the order parameter at free S layer surface $\Delta_{S}$ as a function of angle θ between the $\vec{h}$ and $\vec{n_{x}}$ directions for the case α=β. It is seen that, in the absence of SOI (α=β=0; green line in the figure), $\Delta_{S}$ is independent of angle θ and nearly equal to $\Delta_{S}^{*}\approx 0.4T_{c}.$ This is natural, since, in the absence of spin-active electron scattering, the suppression of superconducting singlet correlations in the S layer depends only on the value of the exchange energy in the F film and does not depend on the direction of its magnetization vector. An increase in the intensity of SOI in the normal layer leads to a nonmonotonic $\Delta_{S}\left(\theta \right)$ dependence. $\Delta_{S}$ increases with θ, reaches a maximum at θ=π/4, and then, decreases, achieving $\Delta_{S}=\Delta_{S}^{*}$ at θ=3π/4.

Figure 3b reveals the reason for the nonmonotonicity of $\Delta_{S}\left(\theta \right)$. With an increase in the angle θ, there is a significant change in the spatial dependencies of the triplet anomalous functions $f_{1}\left(z\right)$ and $f_{2}\left(z\right)$. When θ=π/4, Equation (6) for functions $f_{1}\left(z\right)$ and $f_{2}\left(z\right)$ becomes exactly equal, and the functions turn out to coincide. In this case, the SOI term in Equation (5) consists of two parts with the same sign and having a significant value. Thus, the functions undergo a rapid decrease inside the layer and a sharp jump on the N${}_{SO}$F interface.

At θ=3π/4, functions $f_{1}\left(z\right)$ and $f_{2}\left(z\right)$ are anti-symmetric. In this case, the SOI term in Equation (5) consists of two parts with different signs, which compensate each other. In the particular case α=β, the SOI term totally vanishes. As a result, at θ=3π/4, the triplet correlations penetrating into the S film turn out to be significantly more intense than in the case of θ=π/4. It is this difference that leads to changes in the dependency $\Delta_{S}\left(\theta \right)$ represented in Figure 3a. Thus, at θ=π/4, the normal metal acquires the role of a filter, which shields the superconductor from the ferromagnetic film, that is the presence of SOI leads to a weakening of the spin correlations of electrons initiated by the ferromagnet in the SN${}_{SO}$ sandwich. At θ=3π/4, vectors $\vec{h}$ and $\vec{A}$ are perpendicular to each other, the filtering is absent, and anomalous functions $f_{1}\left(z\right)$ and $f_{2}\left(z\right)$ coincide with them calculated in the absence of SOI, so $\Delta_{S}=\Delta_{S}^{*}$.

It should be noted that there is a natural upper limit to the efficiency of the spin valve $\eta =\Delta_{S}\left(\pi /4\right)/\Delta_{S}^{*}$. This is due to the fact that the value of $\Delta_{S}\left(\pi /4\right)$ is limited by the value of $\Delta_{S}$ calculated in the structure SN${}_{so}$F with h=0 in the F film.

The larger the difference between the coefficients α and β, the smaller the parameter η is. Figure 4a gives the $\Delta_{S}\left(\theta \right)$ dependencies calculated for θ=π/4, different values of parameter β, and α=1. It is seen that, for β=0, the magnitude of $\Delta_{S}$ is larger than $\Delta_{S}^{*}$ and independent of θ. With the growth of the parameter β, the nonmonotonicity in the $\Delta_{S}\left(\theta \right)$ dependence is restored. Figure 4b shows the $\Delta_{S}\left(\beta \right)$ curves calculated for α=0 (blue dotted curve), α=β (red dotted curve), and α=2 (black solid curve). For all values of α, the dependencies show a monotonic growth with parameter β. It can also be seen that the steepest growth of $\Delta_{S}$ is observed in the case of α=β. As discussed above, such a significant increase in $\Delta_{S}\left(\beta \right)$ is due to the best protection of singlet superconducting correlations in the S layer from triplet pairings, which is realized at α=β.

We conducted a study of the proximity effect in the SN${}_{SO}$F structures at sufficiently large thicknesses of the F layer $d_{F}=2\xi$. The calculations showed that the SN${}_{SO}$F multilayer is indeed a spin valve. In it, changing the direction of the magnetization vector of the ferromagnet from parallel to the spin–orbit vector $\vec{A}$ to the perpendicular direction leads to the suppression of the superconducting properties in the structures. This effect is most pronounced when the Rashba and Dresselhaus SOI coefficients are equal to each other. However, with the selected thickness of the F film, the efficiency η of the spin valve effect turned out to be insignificant. Below, we will show that, with a decrease in $d_{F}$, the efficiency of the spin valve effect can be significantly increased. To do this, we will analyze the difference between the $\Delta_{S}\left(d_{F}\right)$ dependencies calculated for α=β and two fixed angles θ=π/4 and θ=3π/4.

## 5. SN${}_{\mathrm{SO}}$F Spin Valve

Reducing the thickness of the F layer should be accompanied by two effects.

The first of them follows from the oscillatory nature of the coordinate dependence of superconducting correlations in a ferromagnet [22,76,77,78]. The combined effect of the boundary conditions (10) and the oscillatory nature of the $f_{i}\left(z\right)$ functions in a ferromagnet impose strict requirements on the shape of the $f_{i}\left(z\right)$ dependencies in the F film. It, in turn, dictates the form of all other spatial variations in the structure, including the sign of the order parameter in its S part.

The second effect is a decrease in the value of the effective exchange energy in the NF bilayer [79,80,81,82]. In it, the electron can spend some time in the N part of the structure, in which the spin ordering is absent. This is equivalent to the action on electrons of the effective exchange energy averaged over the thickness of the FN structure. It is obviously less than the exchange energy in its ferromagnetic part.

**Figure 5:**
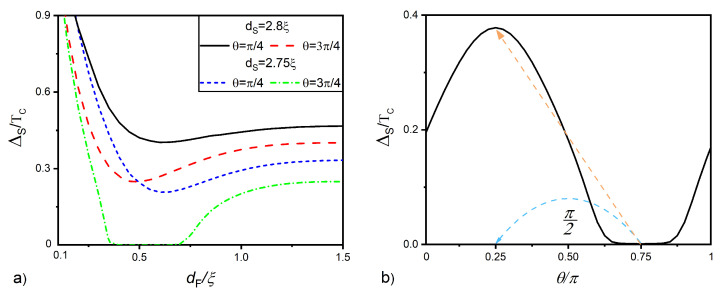
Pair potential $\Delta_{S}$ at the free surface of the S layer versus (**a**) F layer thickness $d_{F}$ at different S layer thicknesses $d_{S}$ and at angles θ=π/4 and θ=3π/4 (**b**) versus magnetization angle θ at $d_{S}=2.75\xi$ and $d_{F}=0.38\xi$. The other parameters are $\alpha =\beta =1,\;/,\;d_{N_{SO}}=0.2\xi_{S},\;/,\;h=20T_{C},\;/,\;T=0.5T_{C},\;/,\;\gamma_{B}=0.3$.

Our calculations confirmed the manifestation of both of these effects in SN${}_{SO}$F structures. Figure 5a shows the dependencies of the magnitude of pair potential $\Delta_{S}$ at the free S layer surface on the F layer thickness $d_{F}$ calculated for angles θ=π/4 and θ=3π/4 and two different S film thicknesses $d_{S}=2.8\xi$ and $d_{S}=2.75\xi .$ For $d_{S}=2.8\xi$, it demonstrates the presence of the local minimum on the $\Delta_{S}\left(d_{F}\right)$ curves for both open (black solid curve, θ=π/4) and closed (dotted red curve, θ=3π/4) SN${}_{SO}$F spin–orbit valves. At the minimum point on the dependence $\Delta_{S}\left(d_{F}\right)$, there is the sign change of the functions $f_{0}$ in the S film (so-called 0-π phase transition). The difference between the black solid and the dotted red curves has a maximum in the vicinity of this 0-π transition.

For $d_{S}=2.75\xi ,$ $\Delta_{S}\left(d_{F}\right)$ exhibits the so-called reentrant behavior [83,84]. In the interval $0.4≲d_{F}/\xi ≲0.8$, there is the complete destruction of superconductivity with its restoration at small $d_{F}≲0.4\xi_{S}$ and large $d_{F}≳0.8\xi_{S}$ F layer thicknesses (see the green dotted curve in Figure 5a calculated for θ=3π/4). Contrary to that, at θ=π/4 (blue dotted line in Figure 5a), superconductivity exists for all values of $d_{F}.$

The origin of this feature corresponds to the spatial oscillations of the pair amplitude *f* in the half-infinite F layer. In the case of the F layer of finite thickness, the oscillatory behavior is limited by the boundary condition on the free surface of the F layer $df_{i}/dz=0$. As result, at the transition between the 0 and π phase of the *f* function on the surface, the system enters the energetically unfavorable state, leading to the decrease or even elimination of the pair potential $\Delta_{S}$ in the S layer.

Figure 5b demonstrates the implementation of the claimed spin valve effect. It gives the dependence of $\Delta_{S}$ on angle θ calculated for $d_{S}=2.75\xi ,$ $d_{F}=0.38\xi ,\;d_{NSO}=0.2\xi_{S},$ $\alpha =\beta =1,\;h=20T_{C},\;T=0.5T_{C},\;\gamma_{B}=0.3.$ It is clearly seen that with the change in the direction of the magnetization vector on π/2 from θ=3π/4 to θ=π/4 (dotted red arrow), the superconducting order parameter changes from $\Delta_{S}=0$ up to the value of $\Delta_{S}\approx 0.4T_{c}\approx 10^{-3}\mathrm{eV}$ (for the case of a frequently used superconductor Nb c $T_{c}\approx 10K$).

## 6. SF’F Spin Valve

Turning to the comparison of the operating modes of the SN${}_{SO}$F and SF’F spin valves, it should be noted that, previously, it was usually assumed that in conventional SF’F spin valves, the coercive force of the upper ferromagnetic film (F) significantly exceeds the coercive force of the lower (F’) layer. As a rule, this was achieved either due to a significant difference in the thicknesses of ferromagnetic films, $d_{F^{\prime }}$ and $d_{F}$, or by organizing the contact of the F layer with the antiferromagnetic material. At the same time, in the theoretical analysis of processes in SF’F spin valves, it was assumed that $d_{F}\gg d_{F^{\prime }}.$

Below, we fixed the thickness of the layer F’, with the value $d_{F^{\prime }}=0.2\xi$ equal to the thickness of a normal film in an SN${}_{SO}$F device. We also assumed that the exchange energies of both magnetic layers are the same ($h=20T_{C}$) and that the difference in the coercive forces of the F’- and F films allows us, at an arbitrary ratio between $d_{F}$ and $d_{F^{\prime }}$, to change the direction of the vector, $\vec{M^{\prime },}$ keeping the direction of the vector, $\vec{M,}$ of the F film fixed. Finally, we selected the thickness of the S layer near the critical value of $d_{S}^{crit}\approx 3\xi$ in order to observe changes in $\Delta_{S}$ in the same range of magnitudes as in the SN${}_{SO}$F device. $d_{S}^{crit}\left(\theta \right)$ is the maximum value of the S film thickness at which superconductivity is still completely suppressed throughout the valve volume and $\Delta_{S}=0.$

The main properties of the SF’F valves can be analyzed in the frame of the same boundary value problem (3)–(11) with the substitution of Equation (5) for that of the F^′^ layer:

(12) $$\begin{array}{c} \begin{array}{c} Df_{0}-if_{1}h=0,\\ Df_{1}-if_{0}h=0,\\ Df_{2}=0,\\ Df_{3}=0; \end{array} \end{array}$$

The F^′^ layer does not protect the superconductor S-wave ordering across the SF’F structures from triplet correlations as effective as the N${}_{SO}$ layer in SN${}_{SO}$F devices. As a result, it turns out that the critical thickness of S layer $d_{S}^{crit}$ in SN${}_{SO}$F structures is significantly smaller in comparison with that of SF’F structures in the case of ferromagnetic ordering of magnetization vectors in F’- and F films.

**Figure 6:**
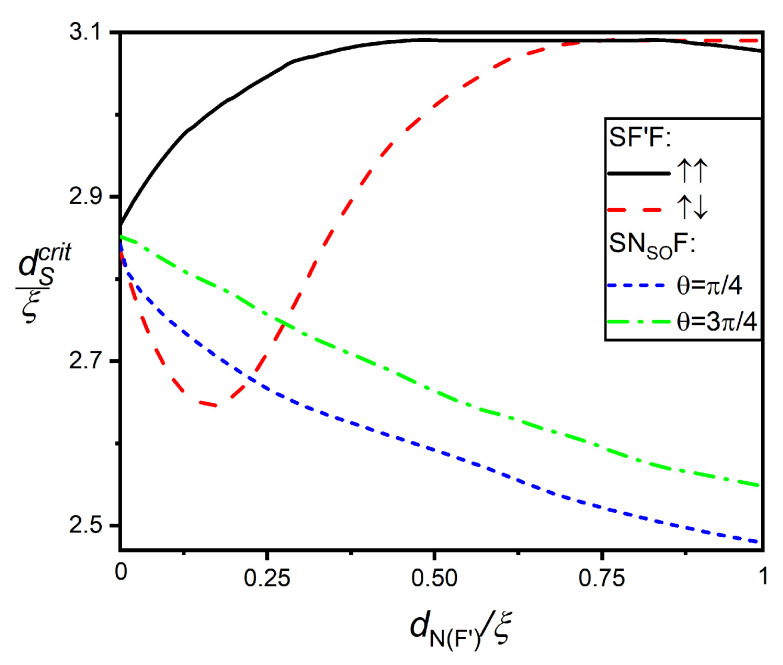
Critical thickness of the S layer $d_{S}^{crit}$ versus thickness of the intermediate layer in $SF^{\prime }F$ structures $d_{F^{\prime }}$ calculated for parallel (solid black curve) and antiparallel (dotted red curve) orientations of magnetization vectors of F- and F’ films and $d_{S}^{crit}$ as a function of $d_{NSO}$ of $SN_{SO}F$ spin valves calculated for α=β=1 and θ=π/4 (dotted blue curve) and θ=3π/4 (dashed green curve). Other parameters are $d_{F}=0.4\xi ,\;h_{F^{\prime }}=h_{F}=20T_{C},\;T=0.5T_{C},\;\gamma_{B}=0.3$.

Figure 6 demonstrates the critical thicknesses of S layer $d_{S}^{crit}$ in SN${}_{SO}$F and SF’F structures as a function of the thicknesses of their middle layer ($d_{NSO}$ or $d_{F^{\prime }}$, respectively) for different orientations of the magnetization vector of the F layer and for a fixed thickness $d_{F}=0.4\xi$. It can be seen that in SF’F spin valves’ $d_{S}^{crit}$ monotonically increases with $d_{F^{\prime }},$ if $\vec{M^{\prime }}$ are parallel to $\vec{M}.$ In the case of the antiparallel orientation of $\vec{M^{\prime }}$ and $\vec{M}$, the values of $d_{S}^{crit}$ decrease from $d_{F^{\prime }},$ reaching a minimum at $d_{F^{\prime }}\approx 0.2$ and, then, monotonically growing with $d_{F^{\prime }}$ increasing. In SN${}_{SO}$F spin valves, $d_{S}^{crit}$ monotonically decreases with increasing $d_{NSO}$ for both values θ=π/4 (dotted blue curve) θ=3π/4 ( dashed green curve) and α=β. The difference between the green and blue curves is due to the better protection of the S-wave pairing in the structure that takes place at θ=π/4.

**Figure 7:**
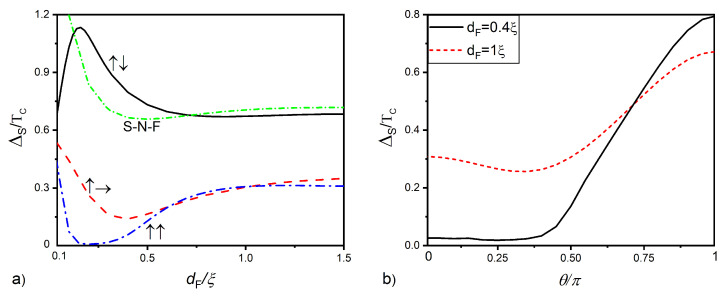
Pair potential $\Delta_{S}$ at the free surface of the S layer of the S-F’-F structure versus (**a**) F layer thickness $d_{F}$ at different directions of magnetization of the F layer θ=0 (blue dashed-dotted curve), θ=π (black solid curve), and θ=π/2 (red dashed curve). The green dashed-dotted curve reveals the same dependence in S-N-F structure with zero exchange energy in the intermediate layer. (**b**) Pair potential $\Delta_{S}$ versus magnetization angle θ at $d_{F}=0.4\xi$ and $d_{F}=\xi$. The other parameters are $d_{S}=3\xi ,\;/,\;d_{F^{\prime }}=0.2\xi ,\;/,\;h_{F^{\prime }}=h_{F}=20T_{C},\;/,\;T=0.5T_{C},\;/,\;\gamma_{B}=0.3$.

Figure 7a shows the order parameter on the free surface of the superconductor $\Delta_{S}$ as a function of the thickness of the F layer for parallel (blue dashed-dotted curve), perpendicular (red dashed curve), and antiparallel (solid black curves) arrangements of the magnetization vectors ${\vec{M}}^{\prime }$ and $\vec{M}$ of the ferromagnetic layers. The green dashed-dotted line is the same dependence for the SNF structure with zero exchange energy in the intermediate layer.

With a parallel $\vec{M^{\prime }}$ and $\vec{M}$ arrangement (blue dashed-dotted curve), both magnetic layers act as a single ferromagnetic region and provide the strongest suppression of the pair potential $\Delta_{S}$. In the thickness range $0,12\xi ≲d_{F}≲0.25\xi$, superconductivity is completely suppressed and restored at larger values of $d_{F}.$ The reason for this effect is the same as in the SN${}_{SO}$F structures discussed above.

With the orthogonal orientation of the vectors $\vec{M^{\prime }}$ and $\vec{M}$ (red dotted line), an effect associated with the formation of long-range triplet correlations with the projection of spin ±1 onto the magnetization vector $\vec{M}$ should be observed. It really takes place inside the interval $0.6\xi ≲d_{F}≲1.0\xi$, where the pair potential $\Delta_{S}$ in the orthogonal state becomes a little bit smaller than in the parallel $\vec{M^{\prime }}$ and $\vec{M}$ arrangement.

In a situation where the vectors $\vec{M^{\prime }}$ and $\vec{M}$ are antiparallel with the growth of $d_{F}$, there is an increasing compensation of the value of the effective exchange energy in the F’F bilayer, so that $\Delta_{S}$ increases with the growth of $d_{F}$. At $d_{F}=d_{F}^{\prime }$, the compensation is maximal, the effective exchange energy is zero, and the F’F sandwich actually ceases to be a ferromagnetic material [85,86,87,88,89,90,91,92,93,94,95,96,97] and acts on the S film as an N’N normal metal. As a consequence, at $d_{F}=d_{F^{\prime }}$, even larger values of $\Delta_{S}$ are achieved in the SF’F spin valve than the S-N-F structure (the green short dotted line) with an ordinary normal metal film instead of an F’ layer. With a further increase in $d_{F}$, the compensating effect of the F’ film weakens, and at $d_{F}≳0.75$, the values of $\Delta_{S}$ in the SF’F valve turn out to be slightly less than in the SNF structure.

Figure 7b reveals spin valve effect in the SF’F structure demonstrating the dependence of the pair potential $\Delta_{S}$ versus the angle θ between the directions of vectors $\vec{M^{\prime }}$ and $\vec{M}$. It has nonlinear behavior with a possible minimum (red dashed line) at intermediate angles due to the presence of long-range triplet correlations, which additionally suppress superconducting order in the S layer.

## 7. Discussion

Our calculations showed that the most-effective control of superconductivity in spin valves SN${}_{SO}$F and SF’F types is achieved at a film thickness F $d_{F}≲0.5-0.7\xi .$

In SF’F spin valves, such control is realized due to a decrease in the effective exchange energy in the F’F bilayer, which occurs when the mutual orientation of the vectors $\vec{M^{\prime }}$ and $\vec{M}$ changes from parallel to antiparallel. The effect is maximal with an equal thickness of layers, but it is difficult to implement. When the thickness of $d_{F^{\prime }}$ is small compared to ξ, fixing the direction of the vector $\vec{M}$ when switching is a difficult task. It should also be noted that, in the case of small thicknesses of ferromagnetic films, the effect of long-range triplet correlations on processes in SF’F structures is small. The boundary condition on the free surface F of the film imposes on the functions $f_{3}$ a solution independent of *z* in ferromagnets. The matching of such a solution with the exponentially decaying behavior of $f_{3}\left(z\right)$ in the S layer is possible only if $f_{3}$ is equal to zero in ferromagnets. The triplet spin valve effect begins to manifest when $d_{F}$ is increased. However, as our calculations showed, with $d_{F}≲\xi$, its effect is insignificant.

The most-effective superconductivity control in SN${}_{SO}$F spin valves is also achieved at thicknesses of N${}_{SO}$ and F films small compared to ξ. Our calculations showed that, at α=β, a π/2 change in the orientation of the magnetization vector of the F film can lead to a switch from the complete absence of superconductivity to its restoration to values realized in SN${}_{SO}$F devices with zero exchange energy in the ferromagnetic layer.

A smooth change in the direction of the vector $\vec{M}$ opens up the possibility of smooth control over changes in the superconducting properties of the S film. Such management is very much in demand when designing neuromorphic devices, which require smoothly tunable elements [12,98,99].

The absence of the second ferromagnetic layer in the SO spin valve may significantly simplify the control actions, since they require modifying the magnetization direction of the only F layer and pave the way for using ferromagnets with a higher coercive force [100], thus facilitating the half-select problem.

At the same time, the development of SN${}_{SO}$F spin valves is constrained by the search for a suitable material in which the Rashba and Dresselhaus SOI simultaneously coexist. It is possible [101] that InSb has such properties. Another possible solution is to localize one of the SOI on the SN or NF interface and the second SOI in the volume of the N metal. In any case, the search for a normal metal with the necessary properties for the SN${}_{SO}$F spin valve is an urgent task of materials science.

## Data Availability

The data presented in this study are available upon request from the corresponding author.
